# Unveiling the hemolymphatic miRNome composition of the schistosomiasis vector snail *Biomphalaria glabrata*

**DOI:** 10.1016/j.crpvbd.2025.100262

**Published:** 2025-04-22

**Authors:** Sarah Dametto, Benjamin Gourbal, Cristian Chaparro, Silvain Pinaud, David Duval

**Affiliations:** a IHPE, Université de Perpignan Via Domitia, CNRS, Ifremer, Université de Montpellier, Perpignan, France; b MIVEGEC, Université de Montpellier, IRD, CNRS, Montpellier, France

**Keywords:** *Biomphalaria glabrata*, Hemocytes, miRNA, Small RNAs, Host-parasite interaction

## Abstract

MiRNAs are single-stranded short noncoding sequences which display crucial roles on gene transcription regulation in many biological processes especially such as embryonic development, cell proliferation or apoptosis. Also, they are recognized for triggering the host’s internal defence mechanisms and immune cell responses thereby playing crucial role in host-parasite interactions. In the present study, a snap-shot of miRNAs, referred to as the miRNome, is described from the hemolymph, the main immune-related compartment of *Biomphalaria glabrata* snails, one of the intermediate hosts of the trematode parasite *Schistosoma mansoni*, the causative agent of schistosomiasis. A high throughput sequencing approach of small RNAs has revealed the presence of 63 miRNAs in the hemolymphatic compartment. Mollusc-specific miRNAs including *bgl-miR-1985-*5p and *bgl-miR-1984-*5p were identified, along with 25 novel miRNAs. Bioinformatic predictions, thanks to multiple software tools, helped us to identify more than 6000 potential miRNA target gene candidates. Among them is BgTEP1, a complement-like factor involved in parasite clearance. Interestingly, this factor appeared to be targeted by a newly identified miRNA, named *bgl-miR-22707-5p*. Our study underscores the inherent diversity of miRNAs in the hemolymph of *B. glabrata* and discusses their potential role in the regulation of the snail’s innate immune response.

## Highlights

• Constitutive hemolymphatic microRNAs from *Biomphalaria glabrata.*
• Characterization of 25 new miRNAs over 63 yet identified.
• More than 6000 potential miRNA target gene candidates identified.
• MicroRNAs potential regulation of immune genes.

## Graphical abstract

**Figure:**
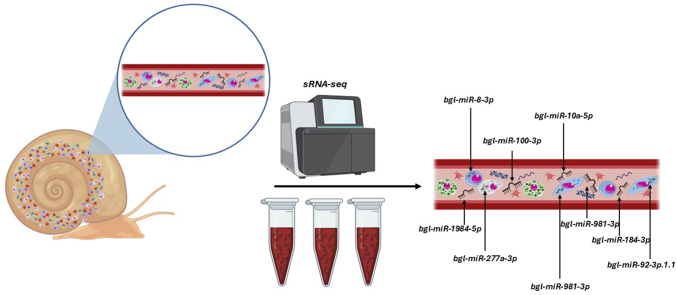

## 1 Introduction

MiRNAs are small regulatory RNAs, ranging from 18 to 24 nucleotides in length, known to regulate gene expression by binding to specific target mRNAs, thereby inhibiting their translation or promoting their degradation (Berezikov, 2011). Since their discovery in 1993 in *Caenorhabditis elegans* (Lee et al., 1993), miRNAs have been extensively studied in various invertebrate organisms. For instance, the initial lin-4 and let-7 miRNAs were identified as key regulators as of the timing and specification of neuronal and hypodermal cell fate during *C. elegans* larval development (Ying et al., 2008). Additionally, certain miRNAs such as mir-184 and mir-277 in *Drosophila melanogaster* are known to play roles in the development and maintenance of the nervous system (Faggins, 2013). Concerning their implications in immunity, numerous reviews have synthetized their functions in the regulation of the immune blood cell response of vertebrates (O’Connell et al., 2010; Chen et al., 2013; Hadj-Moussa et al., 2022) and in innate immune response of invertebrates (Yang et al., 2012; Burgos-Aceves et al., 2018). As an example, miR-100, miR-1984, miR-184 and the miR-9/200/8 families have been highlighted as miRNAs involved in immune-response processes in marine invertebrates (Yang et al., 2012). These miRNAs regulate immune defence and capacities through their own transcriptional regulation, leading to changes in the immune system’s effectiveness (Zhou et al., 2014). In the immune cells of molluscs and arthropods named hemocytes, some miRNAs are conserved and known to be involved in immune response activation through the regulation of pro-inflammatory cytokines (Weitzel et al., 2009). These miRNAs appear to positively regulate phagocytic activity, as seen with miR-184 or miR-190 (Yang et al., 2012; Martín-Gómez et al., 2014), and are involved in processes such as apoptosis, migration and other immune cellular processes, exemplified by miR-92a (Martín-Gómez et al., 2012). In coelomocyte of *Apostichopus japonicas*, miRNAs are involved in regulating immune pathways, miR-133 modulates Toll-like receptor (TLR) signaling by regulating the IRAK-1 protein, resulting in increased phagocytic activity (Lu et al., 2015). Additionally, in the European flat oyster (*Ostrea edulis*) the overexpression of miR-18 (Lee and Hyun, 2014) or miR-335 has been associated with the increase of reactive oxygen species (ROS) levels (Morga et al., 2011, 2012).

*Biomphalaria glabrata* is a Neotropical planorbid snail that transmits in South America and Caribbean islands the trematode parasite *Schistosoma mansoni*, the etiological agent of schistosomiasis (Kokaliaris et al., 2022). Schistosomiasis is considered as a neglected tropical disease, however responsible for almost 200,000 human deaths each year (Lo et al., 2022). Consequently, this parasitic disease remains a major global public health problem despite considerable efforts to prevent and/or treat it. To find new ways to fight and control this disease, it appeared necessary to enhance our current knowledge and understanding of the molecular interactions between *Schistosoma* spp. and their hosts, including especially its intermediate mollusc snail vectors, *Biomphalaria glabrata* (Basch, 1976). *Biomphalaria glabrata* develops some complex innate immune responses to eliminate this trematode parasite. Its internal defence system includes an innate immune cellular and humoral response resulting in parasite encapsulation and killing following the recognition of the parasite by pathogen recognition receptors, the activation of immune signalling pathway and the activation of the cellular immune response in which hemocytes are involved. Indeed, local co-evolution between the two organisms has led to a compatibility polymorphism. It had been observed that the intensity and rate of infection depend on the co-evolution between the two organisms. Théron et al. (2014) showed that with its sympatric strain, BgBRE snails can reach 100% infection rate with 20 miracidia compared to an allopatric strain such as smGUA (Guadeloupe strain) where only 5% of snails were infected. This difference is also evident in the intensity of mother sporocyst (MSp) development, with an average of 3.58 MSp developed with SmBRE and 1 MSp with SmGUA (Théron et al., 2014; Galinier et al., 2017; Mitta et al., 2017; Portet et al., 2019). Comparative approaches, along with genomic, transcriptomic, proteomic and epigenetics studies, have mainly demonstrated strong gene cluster regulation during parasitic infection (Hanington et al., 2010; Coustau et al., 2015; Tennessen et al., 2015; Pinaud et al., 2016; Galinier et al., 2017; Pila et al., 2017; Portet et al., 2017, 2019; Wu et al., 2017; Allan et al., 2019; Mendes et al., 2019; Lu et al., 2020, 2022; Li et al., 2022; Simphor et al., 2024). In this context, while the role of miRNAs in the response of the vertebrate host to exposure to the parasite has been studied (El-Taweel et al., 2022; Hamway et al., 2022), the involvement of miRNAs in the immune response of the intermediate host has been largely neglected (Queiroz et al., 2017, 2020; Alves et al., 2023).

To date, most studies have focused on whole snails rather than hemocytes, probably due to the low abundance of these immune cells and the difficulties associated with their recovery, isolation and enrichment. However, recent advances in massive single-cell sequencing approaches have revealed a remarkable and intricate landscape within hemocytes, key producers of immune effectors and known to play crucial roles in immune defence through encapsulation, phagocytosis and antimicrobial activities (Mitta et al., 2017; Li et al., 2022; Pichon et al., 2022). Moreover, when the miracidium enters the snail host, the parasite comes into direct contact with the hemolymph that bathes all mollusc tissues, as the mollusc has an open circulatory system. The immune response against the parasite is then extremely rapid and in just 3 to 6 hours, the first hemocytes are already activated and arrive in contact with the parasite to eliminate it. This suggests the constitutive presence and very quick regulation of key immune response molecules directly present in the snail hemolymph. Thus, in the present study, we propose to characterize for the first time the miRNome present in the hemolymph compartment of the intermediate snail host *B. glabrata*.

## 2 Materials and methods

### 2.1 Biological material

Snails of the albino strain of *Biomphalaria glabrata* (BgBRE2) were used. This strain was recovered in the field in 1975 from the locality of Recife, Brazil, and maintained since then under laboratory conditions. All individuals lived in tanks filled with pond water at 25 °C with a 12:12 h light/dark cycle and were supplied with fresh lettuce *ad libitum*. To determine miRNA sets by deep sequencing, hemolymph (plasma + hemocytes) of adult snails (sexually mature) with a size ranging from 7 to 12 mm was collected using the retraction defence mechanism (Sminia and Barendsen, 1980) and maintained at −80 °C. Two biological replicates corresponding to different batches were performed, each consisting of hemolymph collected from a pool of 20 individuals. The objective of the present study was to describe the hemolymphatic miRNome from uninfected snails. Using hemolymph, we have both the miRNAs freely or transported into exosomes and/or micro-vesicles.

### 2.2 RNA-seq sequencing

Total RNA from the hemolymph pools was extracted using TRI® reagent (Sigma Life Science, Saint Louis, USA), according to the manufacturer’s instructions. Size selection was performed using the Truseq cDNA kit (Illumina, USA) to obtain only reads below 60 bp, and HiSeq SR 50-bp sequencing was performed by Genome Quebec, Canada.

### 2.3 Small RNA analysis

The sequenced reads were filtered according to the quality score using FASTQC software, and the adapter sequences were trimmed using Trim Galore software on the Galaxy server. Only read sizes of 15–50 nucleotides were selected, and those considered to be of low quality (Phred score < 30) were discarded from the analysis. A very stringent quality score was imposed in this study to remove low-quality sequences due to sequencing errors and to ensure a reliable set of sequences given their small size, particularly in the context of isomiR analysis. All reads sequenced have been analysed by the Kraken tool on the Galaxy server to clean all reads belonging to bacterial and protozoan communities present in the snail microbiota (Supplementary file 1: Table S1).

### 2.4 miRNA analysis

Combined tools for miRNAs identification and their target’s prediction are necessary to prevent any false positive candidates. The pipeline of analysis was used with the *B. glabrata* genome BB02 assembly (BglaB1.7 (Adema et al., 2017) from the VectorBase database) (Supplementary file 1: Fig. S1).

#### 2.4.1 Identification of miRNAs

All 15–50 nucleotide reads were mapped on the *B. glabrata* genome using Bowtie2 (Langmead and Salzberg, 2012) with 1 mismatch permitted and MiRDeep2 Mapper software (v.2.0.0.8.1) (for which no mismatch has been authorized). For the prediction of mature miRNAs, MiRDeep2 (v.2.0.1.2) (Friedländer et al., 2012) and ShortStack (3.8.5) (Axtell, 2013) software were used. MiRDeep2 predicted miRNA read clusters (i.e. precursor elements) with a score ≥ 10 or 100% sequence identity to known miRNAs in the available database (MirBase). While MirDeep2 offers high accuracy (98.6–99.9%) and sensitivity (66–76%), ShortStack enhances the sensitivity and specificity of miRNA identification (Axtell, 2013). ShortStack can annotate miRNA and hairpin-association, with strandedness, small RNA size distribution, phasing, repetitiveness, and quantification. For ShortStack analysis, one mismatch and a size range of 20–24 nucleotides were allowed. miRNA read clusters with near and exact hairpin structure (“N15” (missing part of miRNA∗ (star strand)) and “Y” (considering as a true miRNA) were retained for further analysis. The predictions common to both software packages were retained in addition to the new miRNAs predicted by ShortStack.

#### 2.4.2 miRNAs abundance

Reads per million (RPM) were derived and normalized as counts per million (CPM) using ShortStack, while MiRDeep2 provided the number of unique and total reads for both precursor and mature sequences. The RPM for each miRNA predicted by both MiRDeep2 and ShortStack was calculated using the formula: No. of reads mapped to a mature sequence × 10^6^/Total no. of mapped reads from library. Linear regression analysis is available in Supplementary file 1: Fig. S2. Known miRNAs annotated in the *B. glabrata* genome, available in RFAM (v.14.9) and those identified by Queiroz et al. (2020), were used as input data read sets.

#### 2.4.3 miRNA gene target prediction

miRNA target prediction was conducted using 4 software packages: MiRanda (v.3.3a) (Enright et al., 2003), PITA (v.6) (Kertesz et al., 2007), RNAhybrid (v.2.1.2) (Kruger and Rehmsmeier, 2006) and RNA22 (v.2) (Loher and Rigoutsos, 2012). For MiRanda, only predictions with an alignment score threshold of 140 or higher and a minimum energy threshold of −10 kcal/mol were considered. For PITA and RNA22, predictions were performed using default settings, while for RNAhybrid, a *P*-value cut-off < 0.05 was requested. All target predictions were performed on 3′UTR, Protein Coding Gene (PCG) and 5′UTR. To ensure consistency, clusters of common predictions among all tools were created. MiRanda, PITA, RNA22 and RNAhybrid were used to predict potential targets based on the four commonly used features in miRNA target prediction: seed match, conservation, free energy, and site accessibility. Miranda (script last update: 2020) employs a three-step analysis focusing on seed match as the primary feature, followed by free energy and conservation. PITA (script last update: 2008) prioritizes target-site accessibility but initially applies with the seed match criterion, then evaluates site accessibility through free energy scoring and considers target-site abundance. RNA22 (script last update: 2016) can predict targets in the 3′UTRs, 5′UTRs and PCGs, whereas PITA, MiRanda and RNAhybrid typically focus on 3′UTR target sites. For the 3′UTR targets, only predictions common to all four tools were retained. For the 5′UTRs and PCGs, predictions from MiRanda, PITA and RNAhybrid on the RNA22 were overlapped with RNA22 results to identify shared targets. Using multiple software tools is more particularly beneficial for predicting unconventional targets (Wang et al., 2016; Hassan et al., 2022) or for identifying non-conserved interactions and rare sites (Riffo-Campos et al., 2016).

### 2.5 miRNAs nomenclature

To prevent incorrect annotations, the nomenclature described by Budak et al. (2016) has been adopted. Lettered suffixes indicate closely related mature miRNA sequences derived from different precursors or genomic loci, signifying members of the same miRNA family (e.g*. bgl-miR-216a* and *bgl-miR-216b*). Identical mature miRNA sequences that are encoded by different gene in the genome are differentiated by a number following a dash (e.g. *bgl-miR-87b-1* and *bgl-miR-87b-2*). Additionally, the designations 3p or 5p specify miRNAs originating from the 3′- and 5′-arms of the precursor (Griffiths-Jones, 2004). Several variants by length and/or sequences from the same miRNA are considered as isomiRs and are identified with a number suffix after a dot (e.g. canonical miRNA: *bgl-miR-92b-3p.1*; isomiR1: *bgl-miR-92b-3p.1.1*; isomiR2: *bgl-miR-92b-3p.1.2*) to associate them with their respective canonical miRNA (Neilsen et al., 2012).

## 3 Results and discussion

### 3.1 Small RNAs from the hemolymph of *Biomphalaria glabrata* BgBRE2 strain

To deepen the sequencing, reads from two replicates were combined and analysed. Over 67 million of small RNA reads were sequenced from the snail hemolymph. Of these, 38.8% of reads mapped to *B. glabrata* genome (769,104 unique reads; 26,175,564 count reads). Kraken analysis using bacterial and viral genome databases on the Galaxy server assigned 11.19% (7,567,679 count reads) to bacterial and 2.78% (1,882,966 count reads) to viral sequences. The small RNA sequencing from hemolymph samples revealed a high level of diversity of different types of small RNAs with high-quality scores (PHRED score > 30). Sequences ranging from 15 to 50 nucleotides were retained for small RNA analysis to distinguish between mature miRNAs and their precursors.

The percentage of total mapped reads may seem low and could be attributed to the incomplete genomic assembly and/or SNP between the *B. glabrata* genome and the BgBRE2 strain used in this study. Nevertheless, this result aligns with those obtained by Queiroz et al. (2020) from the same biological model, which reported with 344,624 unique reads out of 1,123,762 total unique reads, representing 30.66% of reads mapped to the reference genome.

Here, 28.32% of the aligned reads were identified as sequences annotated as small RNAs in the *B. glabrata* genome corresponding to 7,414,351 count reads, with 491,924 of these identified as unique reads. Mature miRNAs represent approximately 10% of the aligned reads, with 2,601,481 total reads and 2658 unique reads, making them the least abundant. rRNA and tRNA account for 27% of the identified small-RNAs with 22,625 and 16,949 unique reads, respectively. Nearly 5 million unique reads remain unidentified in detail. Although these reads have been annotated as non-coding RNA in the *B. glabrata* genome, their precise characterization is still lacking. Detailed information on small-RNAs identifications is summarized in Supplementary file 1: Table S1.

### 3.2 miRNAs present in the hemolymph of *B. glabrata* BgBRE2 strain

**Fig. 1:**
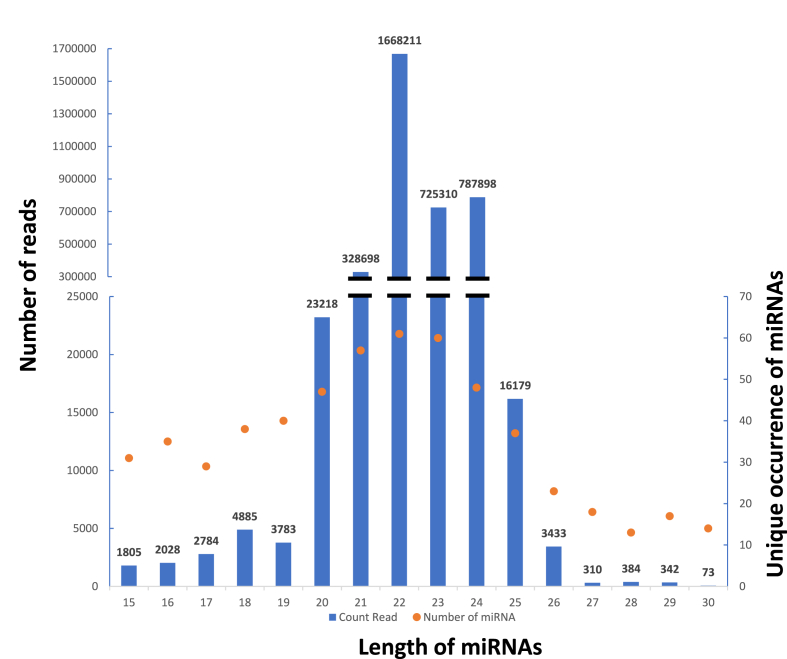
Distribution of count reads (*blue histograms* - left axis) and unique occurrence (*orange points* - right axis) for mature miRNAs by size.

In this study, a total of 3,569,341 count reads (2,601,481 unique reads) aligned to mature miRNAs sequences or pre-miRNAs with a length of 15–30 nucleotides. The length distribution of these reads matches the typical size range for mature miRNAs (Fig. 1). All mature miRNA forms are between 15 and 30 nucleotides, with about 97% of them falling between 21 and 24 nucleotides (3,569,341 count reads).

**Fig. 2:**
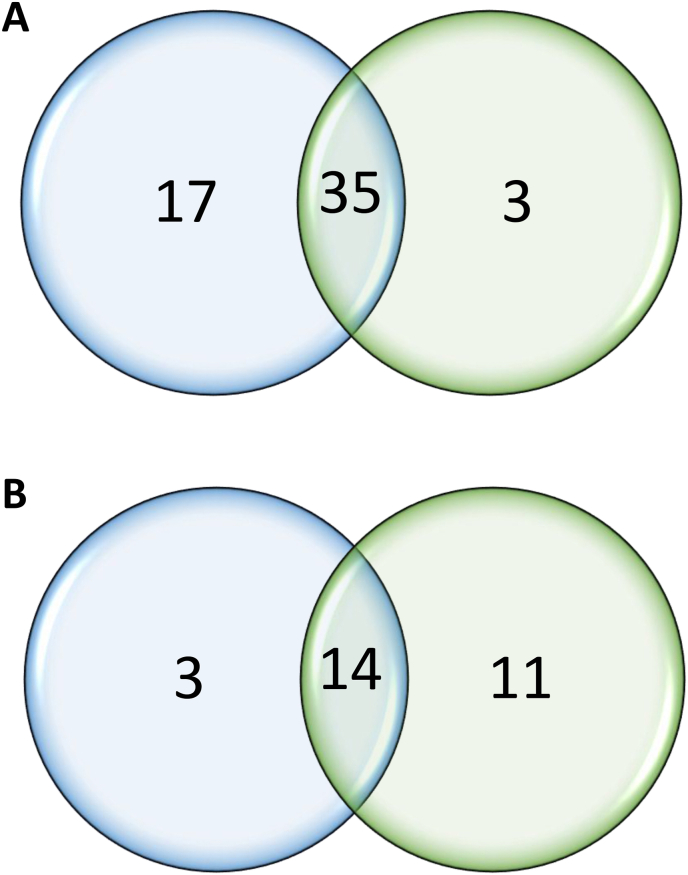
Venn diagram of miRNAs predicted by ShortStack (*green*) and MirDeep2 (*blue*). **A** Known miRNAs. **B**. New miRNAs.

MiRNAs were identified using a comparative tool approach (Berezikov, 2011). Analysis with MiRDeep2 (shown in blue in Fig. 2) identified 52 known miRNAs and 17 new miRNAs. In comparison, ShortStack (shown in green in Fig. 2) detected 38 miRNAs already annotated in the *B. glabrata* genome and 25 new miRNAs (Fig. 2). Both tools commonly identified 35 known miRNAs and 14 new miRNAs. As described in Section 2, predictions commonly from ShortStack and MirDeep2 software (35 known miRNAs and 14 new miRNAs) and the predictions of ShortStack software (3 known miRNAs and 11 new miRNAs) only were retained; the ones strictly predicted only by MirDeep2 were not considered in this study. In total, 63 miRNAs were identified. Of the 25 new miRNAs, 12 did not have corresponding entries in the MiRBase or RFAM databases for any other species with significant *P*-values. Following the consensus nomenclature, these new miRNAs were assigned temporary numbers pending their inclusion in the MiRBase registry. Details of all identified miRNAs are provided in Table 1, Table 2.

**Table 1:** Known miRNAs identified by ShortStack and MiRDeep2.

Mature sequence	Length (bp)	RPM ShortStack	Scaffold ID	Start-End position in scaffold	Gene location	Name
aacccguagaaccgaacuugug	22	17620.732	KE714233	93682–93749	Intergenic	bgl-miR-100-5p
uggacggagaacugauaagggc	22	7504.299	APKA01103429	1551–1691	Intergenic	bgl-miR-184-3p
uucguugucgucgaaaccugccu	23	2777.740	KE712523	35130–35194	Intergenic	bgl-miR-981-3p
ugcccuauccgucaggaacugu	22	1867.472	KE719960	139821–139951	Intergenic	bgl-miR-1984-5p
uaauacugucagguaaagauguc	23	1432.879	KE719932	179103–179262	PCG - Intronic	bgl-miR-8-3p
uaaaugcauuaucugguaucuga	23	1291.194	KE720423	46611–46862	Intergenic	bgl-miR-277a-3p
ugccauuuuuaucagucacugug	23	1261.939	KE719960	136001–136167	Intergenic	bgl-miR-1985-5p
ucccugagaccauaauuugugc	22	932.109	KE714233	104289–104401	Intergenic	bgl-miR-125-5p
ugagguaguagguuguauuguu	22	647.388	KE714233	100237–100421	Intergenic	bgl-miR-let-7-5p
cuuggcacuggcggaauagucac	23	557.631	KE714275	468616–468693	PCG - Intronic	bgl-miR-96a-5p
ccagaucuaacucuuccagcuc	22	466.315	KE714253	178310–178415	PCG - Intronic	bgl-miR-750-3p
ugacuagauccacacucaucc	21	331.940	KE713120	16198–16305	Intergenic	bgl-miR-279-3p
ugagacagugcguccucccuca	22	317.751	KE711897	46379–46444	Intergenic	bgl-miR-1994b-3p
uggcagugugguuagcugguugu	23	266.387	KE720423	58888–59175	PCG - Intronic	bgl-miR-449-5p
aagggagcauccgucgacagu	21	187.180	KE720378	29219–29292	Intergenic	bgl-miR-281-5p
ugaaagacauggguagugagaug	23	169.350	KE720056	15367–15605	PCG - Intronic	bgl-miR-71-5p
ucuuugguuaucuagcuguauga	23	142.338	KE713467	127633–127761	Intergenic	bgl-miR-9-5p
aggcaagauguuggcauagcuga	23	136.975	KE720379	50449–50534	Intergenic	bgl-miR-31-5p
ugagauucaacuccuccaacug	22	104.481	KE714253	170944–171010	PCG - Intronic	bgl-miR-1175-3p
ugagaucauugugaaaacugauu	23	86.785	KE717415	2175–2236	Intergenic	bgl-miR-bantam-1-3p
ugagaucauugugaaaacugauu	23	84.304	APKA01398116	2064–2124	Intergenic	bgl-miR-bantam-2-3p
uuuguucguucggcucgcguua	22	82.595	KE716631	129311–129370	Intergenic	bgl-miR-375-3p
cuaaguacuggugccgcggga	21	70.367	APKA01096956	3003–3106	Intergenic	bgl-miR-252a-5p
agauauguuugauauauuuggug	23	59.521	KE719849	62697–62811	PCG - Intronic	bgl-miR-190-5p
ugagacaguguguccucccuug	22	59.268	KE711897	47019–47082	Intergenic	bgl-miR-1994a-3p
uaauaucagcugguaauccuga	22	23.371	KE711606	757139–757244	PCG - Intronic	bgl-miR-216b-5p
uauuaugcugcuauucacgaga	22	21.202	KE719985	231681–231740	Intergenic	bgl-miR-1993-1-3p
uauuaugcugcuauucacgaga	22	20.296	APKA01101172	817–877	Intergenic	bgl-miR-1993-2-3p
uggcgccguggaaacaucuacc	22	20.132	KE711817	53444–53502	Intergenic	bgl-miR-2722-3p
gagcugccaaaugaagggcugu	22	14.992	KE707076	13220–13338	PCG - Intronic	bgl-miR-745b-3p
uauugcacuuuuccaggccuuu	22	14.813	KE713921	19826–19893	PCG - Intronic	bgl-miR-92a-3p
uugguccccuucaaucaguugu	22	12.689	KE712896	111967–112030	PCG - Intronic	bgl-miR-133-3p
uugugaccguuauaaugggcauu	23	9.880	KE716868	11312–11379	Intergenic	bgl-miR-2001-5p
uagcaccauuugaaaucaguuu	22	9.613	KE715109	101169–101333	Intergenic	bgl-miR-29-3p
gugagcaaaguuucagguguau	22	3.046	KE715168	21952–22013	PCG - Intronic	bgl-miR-87b-2-3p
gugagcaaaguuucagguguau	22	2.704	KE715168	17506–17589	PCG - Intronic	bgl-miR-87b-1-3p
uggaauguaaagaaguauguau	22	1.887	KE712896	141942–142050	PCG - Intronic	bgl-miR-1a-3p
caaugucucugcagugcaauca	22	0.787	KE711726	29962–30064	PCG - Intronic	bgl-miR-33-3p

*Abbreviation*: PCG, Protein Coding Gene.

**Table 2:** Novel miRNAs.

Mature sequence	Length (bp)	RPM ShortStack	Scaffold ID	Start-End position in scaffold	Gene location	Best Hit on miRbase/Queiroz et al. (2020) (*P* < 0.05)	Name
uacccuguagauauccgaauuugu	24	11621.477	KE719220	17337–17405	Intergenic	mle-miR-10a	bgl-miR-10a-5p
aauugcacuucgaccggccugc	22	1262.296	KE713921	19641–19700	PCG - Intronic	bgl-miR-92-3p	bgl-miR-92-3p.1.1
aauugcacuuaccccggccugu	22	654.816	KE713921	19999–20139	PCG - Intronic	bgl-miR-92a-1-3p	bgl-miR-92a-3p.1.2
cauugcacuuuucccggccugu	22	596.380	KE713921	20227–20286	PCG - Intronic	bgl-miR-92a-1-3p	bgl-miR-92a-3p.1.3
uaaugcccccucaaacccuaaa	22	98.077	KE711626	184519–184591	Intergenic	mle-miR-12097-3p	bgl-miR-12097-1-3p
uaaugcccccucaaacccuaaa	22	96.665	APKA01388332	813–876	Intergenic	mle-miR-12097-3p	bgl-miR-12097-2-3p
uaucacagccaguauaccccug	22	52.775	KE720464	153508–153566	PCG - Intronic	lgi-miR-2c	bgl-miR-2c-3p
ugcuccaggauaaagcugcauc	22	15.051	KE707776	2504–2611	PCG - Intronic	NC	bgl-miR-12604-5p
uaucacagccaauaauccccac	22	10.401	KE720464	152823–153010	PCG - Intronic	lgi-miR-2c	bgl-miR-2c-3p.1
uuuuguucggguaguuugaaga	22	10.222	KE714890	12969–13203	Intergenic	bmo-miR-3216	bgl-miR-3216-5p
aaucacaaucauuggauggguu	22	7.964	KE720464	153672–153843	PCG - Intronic	mle-miR-2f	bgl-miR-2f-3p
caucuaccuauccuucuucuuc	22	5.765	KE714719	130732–130795	Intergenic	mle-miR-122104-3p	bgl-miR-122104-3p
uuuaccuacaguuaauacgagug	23	3.937	KE712501	31367–31426	Intergenic	NC	bgl-miR-16599-5p
uuuaccugcagauaauaugaac	22	1.590	KE712501	37204–37258	Intergenic	NC	bgl-miR-21775-3-5p
uuuaccugcagauaauaugaac	22	1.367	APKA01302483	52–107	Intergenic	NC	bgl-miR-21775-2-5p
uuuaccugcagauaauaugaac	22	1.278	APKA01110979	89–144	Intergenic	NC	bgl-miR-21775-1-5p
uugaucaguagcuucaaagaga	22	1.189	KE714890	14783–14841	Intergenic	NC	bgl-miR-22707-5p
cgcgggcguugggggccccacg	22	1.085	KE718106	164257–164314	Intergenic	mle-miR-1986-5p	bgl-miR-1986-5p
uuuggcaccaaagaauucacuga	23	0.862	KE720231	207209–207265	Intergenic	mle-miR-263b-5p	bgl-miR-263b-5p
uggccacgcgcuaucggucucc	22	0.431	APKA01099832	1954–2015	Intergenic	NC	bgl-miR-12705-3p
cgcgacgucacaaccugugggc	22	0.163	KE713001	5787–5883	Intergenic	NC	bgl-miR-22107-5p
agcggaaaggaacuaucucacu	22	0.149	KE713634	6346–6432	Intergenic	NC	bgl-miR-91194-5p
cucaggcugugacguugcaggu	22	0.119	KE710729	100165–100254	Intergenic	NC	bgl-miR-11705-5p
accgguuuacucguc	15	0.104	KE720447	88449–88536	Intergenic	NC	bgl-miR-21902-5p
accgguuuacucguc	15	0.104	KE720447	88449–88536	Intergenic	NC	bgl-miR-21902-3p

*Note*: MiRNAs published by Queiroz et al. (2020) have been recovered and added to our workflow as known miRNAs for our studies. The list of miRNAs is available in the article.

*Abbreviations*: PCG, Protein Coding Gene; NC, Not characterized.

The genomic locations of the 63 mature miRNAs were investigated. Of these, 65% (41 miRNAs) are located in intergenic region, while 22 miRNAs are found within introns of Protein Coding Gene (PCG) sequences. Among the 63 miRNAs, 4 are IsomiRNAs. IsomiRs can be classified into 3′ and 5′ types, with the 3′ isomiR being the most common. When the dominant form is the 5′ isomiR, it can lead to changes in the seed region, also called seed shifting (Berezikov, 2011). Here, the *bgl-miR-92-3p.1.1* has an additional nucleotide at the 3′-end and *bgl-miR-1175-3p.1.1* exhibits trimming at the 3′-end. Additionally, *bgl-miR-92a-3p.1.2* and *bgl-miR-92a-3p.1.3* are isomiRs with nucleotide substitutions in the core and 5′-end, respectively, compared to *bgl-miR-92a-1-3p* as reported by Queiroz et al. (2020), which may result in seed shifting. To adhere to consensus nomenclature, it is proposed to rename these isomiRs to reflect their distinction: *bgl-miR-92a* and *bgl-miR-92a-1* are on the same loci with different mature sequences and should be categorized as isomiRs of *bgl-miR-92a*, designated as *bgl-miR-92a-3p.1.1*.

The three most abundant miRNAs were *bgl-miR-100-5p*, *bgl-miR-10a-5p*, and *bgl-miR-184-3p*, with 744,443, 689,715, and 360,156 count reads, respectively (count per million (CPM) are indicated in Table 3). Together, the 10 mature miRNAs presented in Table 3 accounted for 90.9% of the total read counts among the 63 identified mature miRNAs. Hereafter, we provide descriptions of these 10 more abundant miRNAs of *Biomphalaria glabrata*.

(i) *Bgl-miR-100-5p*: miR-100 is a conserved miRNA found across all bilaterians (Berezikov, 2011). It has been implicated in human cancers, where it influences tumour growth and apoptosis by targeting the mTOR pathway (Zheng et al., 2012; Li et al., 2013, 2015; Sun et al., 2013). Similar functions have been observed in both vertebrates and invertebrates (Yang et al., 2014). Additionally, miR-100 is present in the hemolymph of various invertebrates, including, insects (Dhahbi et al., 2016), oysters (Martín-Gómez et al., 2014) and shrimps (Wang and Zhu, 2017) in which miR-100 has been associated with broader effects on superoxide dismutase, phenol oxidase, apoptosis, and phagocytosis processes (Wang and Zhu, 2017).
(ii) *Bgl-miR-10a-5p*: MiR-10a is a member of the highly conserved miR-10 family, which plays significant role involved in cancer and immune-related processes across vertebrates and invertebrates (Huang et al., 2017). In vertebrates, miR-10a regulates autoimmune diseases like rheumatoid arthritis. Its downregulation leads to the degradation of IκB and activation of NF-κB by targeting IRAK4, TAK1 and BTRC (Mu et al., 2016). In invertebrates, miR-10a has been detected in the hemocytes of *Ostrea edulis* following infection with the protozoan parasite *Bonamia ostreae* (Martín-Gómez et al., 2014) and in *C. gigas* in response to bacterial infections or heat stress (Zhou et al., 2014).
(iii) *Bgl-miR-184-3p*: In vertebrates, miR-184 regulates immune response by reducing the expression of pro-inflammatory cytokines (Weitzel et al., 2009). In *Drosophila melanogaster*, miR-184 is crucial for the development and maintenance of post-embryonic nervous systems (Li et al., 2011). In the shrimp *Marsupenaeus japonicus*, miR-184 is involved in both cellular immunity (such as phagocytosis and apoptosis) and humoral immunity (e.g. phenoloxidase activity) (Yang et al., 2012). In the pea aphid, miR-184 regulates the JNK pathway following bacterial challenges, a pathway known for mediating and controlling phagocytosis, prophenoloxidase (PPO) activation and ROS metabolism (Ma et al., 2020). In *B. glabrata*, *bgl-miR-184-3p* is one of the most abundant and may act as a regulator of apoptosis and proteolysis processes (Chen and Stallings, 2007; Queiroz et al., 2020).
(iv) *Bgl-miR-981-3p*: In *Drosophila*, miR-981 acts as a negative regulator of antibacterial defences by inhibiting the expression of the antimicrobial peptide diptericin expression within the Immune Deficiency (IMD) pathway (Li et al., 2017; Lu and Chtarbanova, 2022). In the shrimp *Penaeus vannamei*, miR-981 modulates the expression of C-type lectins expression in hemocytes, playing a role in the antiviral response against WSSV (White Spot Syndrome Virus) (Shekhar et al., 2019). In *Aedes aegypti*, miR-981 regulates importin β-4, which controls the translocation of AGO1 and its carried miRNAs between the nucleus and the cytoplasm during infection with the endosymbiotic gram-negative bacteria *Wolbachia* (Hussain et al., 2013).
(v) *Bgl-miR-1984-5p*: miR-1984 is recognized as a mollusc-specific miRNA (Im and Kim, 2019) and has also been found occasionally in insects (Zha et al., 2016). It was first described in the gastropods *Lottia gigantea* and *Haliotis rufescens* (Wheeler et al., 2009). In *C. gigas*, miR-1984 is known to play a role in immune response, as it is present in hemocytes and involved in redox regulation and energy metabolism (Zhou et al., 2014; Zhao et al., 2016). In *B. glabrata*, *bgl-miR-1984-5p* is regulated during developmental processes (Queiroz et al., 2020) and upregulated following *Schistosoma* infection in *B. tenagophila* (Alves et al., 2023).
(vi) *Bgl-miR-8-3p*: Commonly observed in both vertebrates and invertebrates, miR-8 is involved in neural development, cell cycle regulation and cell differentiation (Trümbach and Prakash, 2015). In *Drosophila*, Bolin et al. (2016) have shown that loss of miR-8 can protect cells from apoptosis following UV irradiation, and it may also regulate apoptosis in shrimp (Yang et al., 2012). Finally, miR-8 is frequently found in hemocytes of various invertebrates (Martín-Gómez et al., 2014).
(vii) *Bgl-miR-277a-3p*: In *D. melanogaster*, mir-277 is involved in the development and maintenance of the post-embryonic nervous systems (Faggins, 2013). In mosquitoes, CRISPR-Cas9 knockout of mir-277 results in defects in lipid storage and ovarian development (Ling et al., 2017). It was also identified in shrimp hemocytes, where it targets FAD-dependent oxidoreductase (Shekhar et al., 2019). In *B. glabrata* snails, a high level of *bgl-miR-277a-3p* has been observed (Queiroz et al., 2020), suggesting potential roles in endogenous biological processes, immune functions or adaptation to environmental stress.
(viii) *Bgl-miR-92-3p.1.1*: The miR-17-92 family is conserved across all metazoans. Dysregulation of its members can lead to lymphoproliferative diseases and systemic autoimmunity in vertebrates by enhancing the activation, proliferation and survival of T and B cells (Chen et al., 2013). The cluster miR-17-92 includes multiple isomiRs with cancer-regulatory functions in vertebrates (Tong et al., 2012). In oysters, miR-92 regulates the proliferation and development of immune cells by targeting G-protein expression following infection with *Bonamia* sp. (Martín-Gómez et al., 2014). In shrimp, miR-92 levels are modulated in response to apoptotic signals, either being down- or upregulated depending on the context (Yang et al., 2012). In our study, *Bgl-miR-92-3p.1* and one of its isomiRs *bgl-miR-92-3p.1.1* were highly abundant in the hemolymph (Supplementary file 2) assuming for a potential involvement in immune response.
(ix) *Bgl-miR-1985-5p*: Like miR-1984, miR-1985 is also known as a mollusc-specific miRNA (Wheeler et al., 2009). This miRNA has been found to be highly expressed in the hemocytes of *Mytilus galloprovincialis* and has been shown to target cyp-like proteins involved in immune responses against pathogens (Moreira et al., 2020). In *B. glabrata*, *bgl-miR-1985-5p* has been detected in whole snails (Adema et al., 2017; Queiroz et al., 2020) and is also differentially regulated following *S. mansoni* infection (Alves et al., 2023).
(x) *Bgl-miR-125-5p*: miR-125 is highly conserved across the animal kingdom and regulates key processes such as apoptosis, innate immunity, inflammation and hematopoietic differentiation in vertebrates (Martín-Gómez et al., 2014). In the mud crab, miR-125 is expressed in hemocytes and its levels increase following pathogen infection. Knockdown studies of miR-125 reveal that it positively regulates phagocytosis and apoptosis (Qian et al., 2024). In comparison to the study by Queiroz et al. (2020), which explored the miRNome of *B. glabrata* in whole snails, our study reveals that *bgl-miR-981-3p* is more abundant in the hemolymph, with 174,273 unique reads compared to 153,740 count reads in whole snails without shells.

**Table 3:** Abundance of miRNAs by counts per million (CPM): A list of the 10 most abundant miRNAs in the snail immune compartment. The quantification of miRNA abundance using CPM provides insight into their relative representation in the mollusc hemolymph.

miRNA	Counts per million (CPM)
bgl-miR-100-5p	17620.73
bgl-miR-10a-5p	11621.47
bgl-miR-184-3p	7504.29
bgl-miR-981-3p	2777.74
bgl-miR-1984-5p	1867.47
bgl-miR-8-3p	1432.87
bgl-miR-277a-3p	1291.19
bgl-miR-92-3p.1.1	1262.29
bgl-miR-1985-5p	1261.93
bgl-miR-125-5p	932.10

### 3.3 miRNAs gene target prediction

**Fig. 3:**
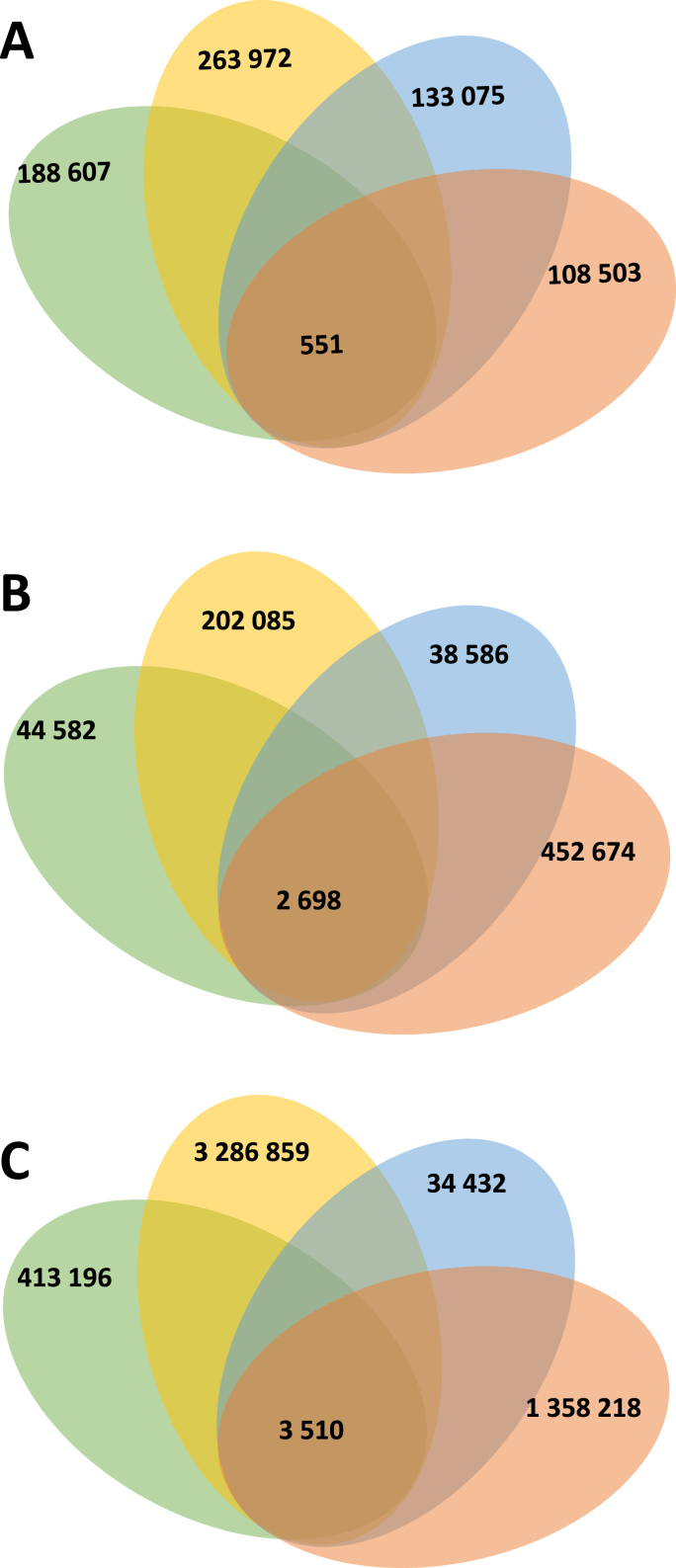
Venn diagram of miRNAs predicted hits on mRNA target. One hit corresponds to the mapping of one miRNA for one mRNA predicted target. The software tools MiRanda (*green*), PITA (*yellow*), RNA22 (*blue*) and RNAhybrid (*orange*) were used. **A** Target’s predictions on 3′UTR. **B** Target’s predictions on 5′UTR. **C** Target’s predictions on Protein Coding Gene (PCG).

**Fig. 4:**
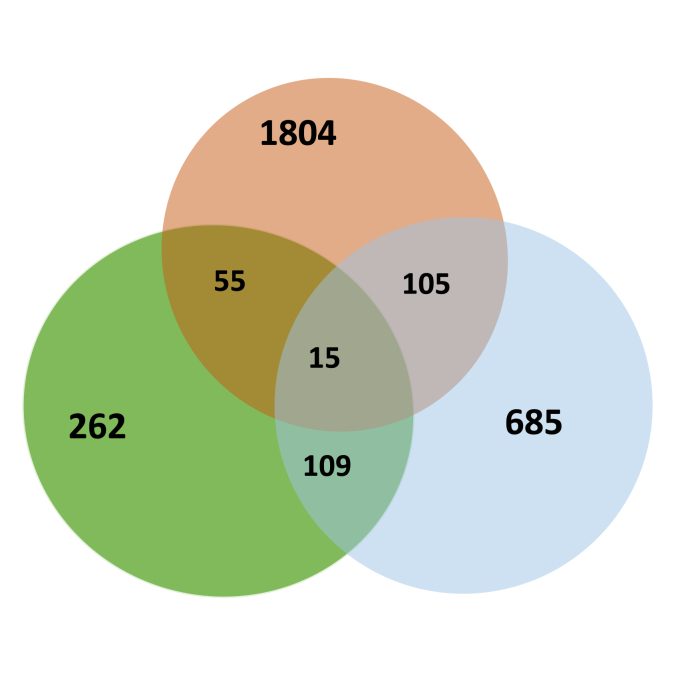
Venn diagram of occurrence of gene targeted by miRNAs on three localisations: 3′UTR (*green*); 5′UTR (*orange*); and Protein Coding Gene (PCG) (*blue*).

For identifying miRNA gene targets, 3′UTR and 5′UTR regions and Protein Coding Gene (PCG) were collected from GFF3 annotation files of *B. glabrata* genome. To improve prediction accuracy and minimize false positives, it is advisable to combine results from multiple prediction tools (Akhtar et al., 2019). Therefore, we employed a multi-tool approach using four prediction software tools: MiRanda, PITA, RNA22 and RNAhybrid. More than 6759 potential miRNA target genes were predicted (Fig. 3; Supplementary file 1). Among these, 551 hits were identified by all prediction tools and correspond to 441 genes for which predicted miRNA-target duplexes are located on the 3′UTR localisation (Fig. 3A). Interestingly, about 2698 hits are presents on the 5′UTR and correspond to 1979 snail genes (Fig. 3 (b)). An even larger number of targets were predicted for the PCG with 3510 hits corresponding to 914 genes (Fig. 3C). In total, 3078 unique miRNAs downregulate genes were identified. Interestingly, some mRNAs are targeted along their entire length by various miRNAs. Specifically, 15 target genes were identified with multiple hits within their 3′UTR, 5′UTR and PCG regions. These hits were contributed by the same miRNAs at different locations or by different miRNAs (Table 4 and Fig. 4).

**Table 4:** Fifteen genes (BB02 and laboratory internal annotation) predicted to be targeted on their total length (UTRs and Protein Coding Gene part) by miRNAs.

BB02 Genome Gene ID	Gene annotation from IHPE Laboratory
BGLB000051	Baculoviral IAP repeat-containing protein 2-like isoform
BGLB000786	ATP-binding cassette sub-family D member 2-like
BGLB000846	Not annotated
BGLB000876	Ras guanine nucleotide exchange factor L-like
BGLB001150	Receptor-type tyrosine-protein phosphatase kappa-like isoform
BGLB001225	Transcriptional activator Myb-like isoform
BGLB016762	Synaptotagmin 7
BGLB017474	Bridge-like lipid transfer protein family member 1 isoform
BGLB020718	Integrator complex subunit 13-like
BGLB022562	Verprolin-like
BGLB027526	Adhesion G-protein coupled receptor D1-like
BGLB028227	D(2) dopamine receptor A-like isoform
BGLB030287	Ankyrin repeat and KH domain-containing protein 1-like
BGLB036645	RWD domain-containing protein 2A
BGLB038139	Gem-associated protein 5-like

An enrichment analysis showed an expected result such as no up- or under-biological, molecular or cellular function are more regulated by miRNAs. We decided to investigate in the literature the biological implications of the 15 targeted genes. All miRNAs targeting this cluster of genes are detailed in Supplementary file 1: Table S4. One of these genes (BGLB000846) is not annotated in the genome. The 14 remaining genes can be categorized into 7 groups.

The first group of gene pertains to protein interaction, specifically the RWD Domain Containing 2A (RWDD2A-BGLB036645). This gene encodes proteins with Ring finger-domains, WD repeat-, and Dead-like helicases (Doerks et al., 2002). While their functions are not well established, it is suggested to be involved in protein interactions (Puthiyedth et al., 2016) and may serve as a substrate for E2 dependent ubiquitination (Lorick et al., 1999).

Two genes (BGLB001225 and BGLB038139), targeted by the *bgl-miR-92a-3p* family, *bgl-miR-9-5p*, or by *bgl-miR-bantam-3p*, constitute the second class associated with transcriptional regulation functions. For example, Gem-associated protein 5 (Gemin5-BGLB038139) identified as a large tryptophan-aspartic acid (WD) repeat protein serves as an RNA-binding protein to deliver small nuclear RNAs to the survival of the motor neurons complex (Gubitz et al., 2002). While its role in the snRNPs (small nuclear ribonucleoproteins) biogenesis is well characterized, recent studies have also highlighted a function as a modulator of translation activity (Pacheco et al., 2009). The second gene, BGLB001225, encodes a Myb transcriptional protein homologous to *b-myb* in vertebrates. This protein is recognized for its role in DNA replication in mitotically dividing larval brain cells and endocycling larval fat body cells in *D. melanogaster* (Davidson et al., 2005). Its role in the JAK/STAT pathway, which mediates hemocytes hyperproliferation and cellular differentiation in hematopoietic organs, has been demonstrated in *D. melanogaster* and mammals (Graf, 1992; Davidson et al., 2005).

Signal transduction can be likened to a game of “telephone” between cells where receptors binding to chemical messengers as circulating hormones and neurotransmitters, pass on the information to a series of intracellular middlemen that ultimately pass on the orders to the final executors (Linder and Gilman, 1992). In our study, miRNAs such *Bgl-miR-9-5p*, *bgl-miR-2c-3p*, *bgl-miR-449-5p* or *bgl-miR-12604-5p* may regulate the mRNA of several genes implicated in this signalling. One notable gene involved in signal transduction is the receptor-type tyrosine-protein phosphatase к (PTPRK-BGLB001150). The tyrosine phosphatases family, including PTPRK, is present across all vertebrate taxa and in the invertebrate chordate *Ciona intestinalis* (Chen et al., 2017). This gene, targeted by several miRNAs including *bgl-miR-2c-3p* and *bgl-miR-12604-5p*, play a critical role in cell adhesion. Loss-of-function in PTPRK has been linked to decreased junctional integrity in mammary epithelial cells (Young et al., 2021) and is particularly significant in the nervous system (Craig and Brady-Kalnay, 2015). Additionally, PTPRK directly regulates the signal transducer and activator of transcription 3 (STAT3) in human’s nasal KT/T-cell lymphoma leading to an increase in patient mortality (Chen et al., 2015). A second gene, BGLB027256, potentially regulated by *bgl-miR-2001-5p*, *bgl-miR-71-5p*, *bgl-miR-let-7-5p*, and *bgl-miR-9-5p*, is adhesion G-coupled protein receptor D1-like (GPCR). This receptor is known for its role in signalling against pathogens and has been implicated in vertebrate immune responses (Wang, 2018). GPCRs are upregulated following exposure to *S. mansoni* in susceptible (11 GPCRs) and resistant (52 GPCRs) snails, highlighting their importance in parasite-snail interactions (Lu et al., 2022). A third gene, BGLB028227, is a (D2) dopamine receptor A-like, a member of the rhodopsin-like GPCR family. This receptor regulates adenylyl cyclase and modulates calcium and potassium channels in cells (Mustard et al., 2005). Phylogenetic analysis shows that in *B. glabrata* D2 receptor is closely related to the those of *Aplysia california* and *Sinonovacula constricta*. In these species, the dopamine 2 receptor has been involved in immune responses to bacterial challenges by modulating SOD (superoxide dismutase) and CAT (catalase) activities (Niu et al., 2019). The last gene involved in signal transduction is an ankyrin repeat and KH domain-containing protein 1 (ANKHD1-BGLB030287), targeted by *bgl-miR-449-5p* but also *bgl-miR-2c-3p*. ANKHD1 contains an ankyrin repeat domain that mediates protein-protein interactions in signalling pathways such as JAK/STAT (Müller et al., 2005), Hippo (Sansores-Garcia et al., 2013), and PINK/PARKIN (M. Zhu et al., 2015b) as well as a KH (K-Homology) domain that binds RNAs, miRNAs or single-stranded DNA (Mullenger et al., 2023). The interaction between miRNAs and circ-ANKHD1 has been investigated revealing that circ-ANKHD1 inhibits miR-27a-3p, which positively regulates SFRP1 (Secreted Frizzled Related Protein 1) expression in granulosa cells in ovarian sows. This modulation promotes granulosa cell proliferation (Li et al., 2021).

Another gene revealed to be targeted by miRNAs (*bgl-miR-11705-5p*, *bgl-miR-449-5p*, and *bgl-miR-71-5p*) corresponds to an integrator complex subunit 13 (BGLB020718). Integrator subunits (INTSs) are a metazoan-specific protein family comprising 15 subunits (Kirstein et al., 2021), that play crucial roles in biological processes such as the cleavage of the extended 3′-end of Uridine-rich small nuclear RNAs, essential for the biogenesis of spliceosomal snRNPs and the 3′-end formation of enhancer RNA (Barbieri et al., 2018). While its role in transcriptional regulation is well documented, its impact on cellular homeostasis, cell proliferation and apoptosis in hepatocellular carcinoma in humans (HCC) has also been highlighted (Wang et al., 2024). Additionally, its association with EGR1/2 TFs, which regulate enhancer regions during the differentiation of progenitor cells into monocytes and macrophages, underscores its significance (Barbieri et al., 2018). This class of cellular functions also includes another gene, BGLB000051, which corresponds to a BIRC2, a member of the anti-apoptotic gene family. This encoded protein is composed of three domains: Baculovirus Inhibitor of Apoptosis Repeat (BIR), Really Interesting New Gene (RING) and Caspase Recruitment Domain (CARD). Studies have highlighted BIRC2’s role in inhibiting apoptosis by interfering with the activation of caspases (Yang and Li, 2000). Several miRNAs are known to target this gene as miR-29c in the cerebral ischemia/reperfusion in rats (Lingjie et al., 2021), or miR-5195-3p in glioma cells in humans (Yang et al., 2020). In our study, BIRC2 is targeted by multiple miRNAs, such as *bgl-miR-bantam-1-3p*, *bgl-miR-bantam-2-3p*, *bgl-miR-let-7-5p*, *bgl-miR-33-3p*, and *bgl-miR-71-*5p. Interestingly, during schistosomiasis, the vertebrate host exhibits a high level of BIRC2 expression during infection in lung endothelial cell (Oliveira et al., 2021). The last gene in this functional category is a ras guanine nucleotide exchange factor L-like (Ras GEF-BGLB000876). Ras is a small G-protein primarily involved in assembling intracellular downstream cell signalling pathways, including those related to proliferation, differentiation, apoptosis, senescence and metabolism (Schlessinger, 2000). GEFs are key regulatory elements in the RAS-GDP/GTP exchange process (Hennig et al., 2015). Activation of GTP triggers a signal transduction cascade by the extracellular signal-regulated kinase (ERK) and mitogen activate protein kinase (MAPK). The Ras/MAPK pathway influences several biological processes, such as the Insulin/IGF-1 signalling (ISS) pathway, the mTOR pathway involved in longevity in both invertebrates and vertebrates, and the AMPK (AMP-activated protein kinase) pathway, a major regulator of energy metabolism, stress resistance and proteostasis (Slack, 2017).

The verprolin protein (BGLB022562) is targeted by a single miRNA, *bgl-miR-9-5p*, across its entire length (5′UTR, PCG and 3′UTR). This protein is involved in cellular processes and more precisely in cellular migration. Verprolin is essential for actin polymerization during polarized growth and endocytosis. In invertebrates, only one verprolin gene has been identified, compared to three in vertebrates, i.e. WASP-interacting protein (WIP), glucocorticoid-regulated gene product (CR16) and WIP-related (WIRE) (Aspenström, 2005).

Another functional class identified through our predictions relates to intracellular lipid transport. Lipids are essential for the homeoviscous adaptation (HVA) enabling cells to adjust to developmental, physiological and environmental changes. Two genes, BGLB017474 and BGLB00786, correspond to a bridge-like lipid transfer protein member 1 (BLTP1) and ATP-binding cassette sub-family D respectively. The BLTP protein family has been characterized for its ability to connect two organelle membranes facilitating non-vesicular lipid transfer *in vitro* (Neuman et al., 2022). BLTP1 is recognized by the HUGO Gene Nomenclature Committee and is found in both vertebrates (known as *KIAA1109* in humans) and in invertebrates (as *ldp-3* in *C. elegans* and *tweek* in the genus *Drosophila*). In *C. elegans*, *ldp-3* gene has been highlighted through CRISPR-GFP studies as a crucial link between plasma membrane and the endoplasmic reticulum for lipid trafficking (Pandey et al., 2023). The second gene in this group, ABCD2-BGLB000786, is an ATP-binding cassette transporter and targeted by *bgl-miR-8-3p*. ABCD family transporters are known to be located in peroxisomal compartment and play a role in fatty acid metabolism by importing proteins (Ford and Beis, 2019; Parreira de Aquino et al., 2021). Studies have showed that depletion of ABCD2 lead to oxidative stress (Fourcade et al., 2009). Moreover, ABCD2 appears to share a similar function with ABCD1 in the import/catabolism of Very Long Chain of Fatty Acid (VLCFA) such as C26:0 (Hexacosanoic acid), where an excess can result in the production of ROS (Fourcade et al., 2010).

The last class of gene highlighted in our study pertains to immunity-related gene functions. One such gene is Synaptotagmin 7 (BGLB016762), which is predicted to be targeted by multiple miRNAs, including *bgl-miR-981-3p*, *bgl-miR-2c-3p*, and *bgl-miR-9-5p*. Notably, *bgl-miR-9-5p* is predicted to target Synaptotagmin 7 on both the 3′UTR and PCG regions. The Synaptotagmin family has been studied for their role in inhibition of cytokine secretions and phagocytosis in macrophages (Du et al., 2017). Additionally, in human thyroid cancer cells, it plays a role in regulation (cell migration and autophagy, processes regulated by miRNA-363-3p) (Zhang et al., 2024).

In general, most of the genes targeted by miRNA characterized in hemolymphatic compartment are involved in immune pathways*. Bgl-miR-100-5p*, *bgl-miR-10a-5p*, and *bgl-miR-1985-5p* target several glycosidase molecules found in the snail’s hemolymph, which can bind to oligosaccharides (such as mannose and galactose as terminal surface carbohydrates) present on the tegument of schistosome sporocysts (Zelck, 1999). Interestingly, the predicted miRNAs could regulate immune genes including toxins like biomphalysins known for their lytic activity against the membrane of the parasite *S. mansoni* (Galinier et al., 2013; Pinaud et al., 2021). Our predictions suggest that several genes encoding immune factors such as interleukin receptors (BGLB40335, BGLB31074, and BGLB007388) who are humoral factors and are targeted by *bgl-miR-1985-5p*, *bgl-miR-981-3p*, *bgl-miR-449-5p* or *bgl-miR-184-3p* in diverse locations (3′UTR, PCG, and 5′UTR). The interleukin factor has been directly associated with the ability of snail hemocytes to kill *S. mansoni* sporocysts (Granath Jr. et al., 2001). Moreover, others immune molecules such as toxins could be targeted by miRNAs. For instance, Biomphalysin 11 (BGLB000108) is targeted by *bgl-miR-184-3p* and *bgl-miR-10a-5p*; Biomphalysin 19 (BGLB000001) is targeted by *bgl-miR-279-3p*; Biomphalysin 18 (BGLB000010) is targeted by *bgl-miR-216-5p*; and Biomphalysin 17 (BGLB000119) is targeted *bgl-miR-11705-5p*. These miRNAs specifically target biomphalysin genes at their 5′UTR. These toxins are known to have cytotoxic activity on the sporocyst, and to be expressed in immune-competent cells (Galinier et al., 2013; Pinaud et al., 2021). Given that *B. glabrata* genome is not fully assembled, particularly for highly polymorphic and diversified multigenic molecules, RNA-seq studies which have been instrumental in reconstructing these molecules’ sequences specific to the BgBRE strain from Recife, Brazil (Dheilly et al., 2015), and in identifying scaffolds in *B. glabrata* genome BB02 (Duval et al., 2020), were used to identify if these immune molecules could be targeted by miRNAs.

### 3.4 Did FREP and TEP are targets for miRNAs?

FREPs (fibrinogen-related proteins) are a highly diversified family of immune pathogen recognition receptors (PRRs). The combination of one or two N-terminal immunoglobulin superfamily (IgSF) domain with a C-terminal fibrinogen-related (FBG) domain defines FREP molecule. Their roles in binding the parasite and leading the immune response for the host have been described and characterized (Leonard et al., 2001; Hertel et al., 2005; Hanington and Zhang, 2011; Galinier et al., 2017; Portet et al., 2017). Co-immunoprecipitation experiments have raised immune complex interactions that associate three partners: FREPs of *B. glabrata*, SmPoMucs of the parasite (polymorphic mucins) and TEPs (thioester-containing protein) molecules (Roger et al., 2008a, 2008b, 2008c; Portet et al., 2017). This antiprotease has been characterised to be involved in innate immune response of *B. glabrata* against the *S. mansoni* (Portet et al., 2018; Pinaud et al., 2019; Duval et al., 2020). Genes from the multigenic families FREPs and TEPs are not well annotated and fully assembled in *B. glabrata* genomes. Therefore, the domains of FREPs and TEPs molecules are predicted to be regulated by miRNAs. Notably, the immunoglobulin domain (IgSF), MAM domain, and LDL-receptor class A related to alpha 2-macroglobulin appear to be targeted by miRNAs such as *bgl-miR-2001-5p*, *bgl-miR-29-3p*, *bgl-miR-12707-5p*, and *bel-miR-31-5p*.

Thus, to assess whether miRNAs might target some members of the BgTEP and FREPs families, we selected their full-length assembled sequences, including the 5′UTR and 3′UTR if this information was available (Duval et al., 2020) (Table 5; Supplementary file 1: Table S2) for inclusion in the present analysis.

**Table 5:** Thioester-containing proteins (TEPs) targeted by miRNAs in hemolymph of *Biomphalaria glabrata* (PCG: Protein Coding Gene).

TEP name	Nos of localisation	miRNA	Localisation
bgA2M	5	bgl-miR-22707-5p	PCG
bgC3-2	1	bgl-miR-252a-5p	PCG
bgC3-2	3	bgl-miR-31-5p	PCG
bgTEP1	2	bgl-miR-22707-5p	PCG
bgTEP2	3	bgl-miR-252a-5p	PCG
bgTEP3	4	bgl-miR-22707-5p	PCG
bgTEP3	3	bgl-miR-8-3p	PCG
bgTEP3	10	bgl-miR-96a-5p	PCG
bgTEP4	3	bgl-miR-216b-5p	PCG
bgTEP4	8	bgl-miR-96a-5p	PCG

Members of the BgTEP family are targeted by several miRNAs, including the newly described *bgl-miR-22707-5p.* Some miRNAs exhibited multiple hits towards the same TEP gene; for example, *bgl-miR-96a-5p* had 10 hits targeting the PCG of BgTEP3 (Table 5). Among the FREPs sequences referring to BgBRE (strain from Recife, Brazil) (Supplementary file 1: Table S3), curiously, no common miRNAs were predicted to target FREPs by the four tools. This result may be explained by three hypotheses: (i) FREP expression may not be regulated by miRNAs; (ii) FREP may be regulated by another gene expression regulator; or (iii) miRNAs implicated in FREP regulation have not been detected in this study.

To go further, a comparative small RNA sequencing of hemocytes from different *B. glabrata* snail strains, infected or not, could provide valuable insights into the roles of miRNAs in host-pathogen interaction, similar to the approaches conducted for *B. tenagophila* highlighting the differential expression of miRNAs between infected/not infected but also between resistant/susceptible strains (Alves et al., 2023). Another prospect is that in response to the miRNAs produced by the hosts, the parasites themselves produce small RNAs to interfere potentially with host immunity (Meningher et al., 2016, 2020). Indeed, in the literature, it has been demonstrated that parasites, especially the intracellular or endo-parasites, can also utilize miRNAs to manipulate gene expression of their hosts, either by secreting their own miRNAs into host cells or by hijacking the host’s miRNAs for their benefit (Weiberg et al., 2013; Cui et al., 2019; Villares et al., 2020). For example, the intracellular parasite *Nosema ceranae* releases exogenous miRNAs that could target host mRNAs in honeybee cells (Fan et al., 2022). Surprisingly, similar strategies have been reported for endoparasites, i.e. *Trichuris suis* (Entwistle and Wilson, 2017), miRNAs and miRNAs from exosomes vesicles secreted by the nematode *Heligmosomoides polygyrus* have been detected in *Sus scrofa domesticus* (pig) (Hansen et al., 2015) and mouse cells, respectively (Buck et al., 2014). Interestingly, the miRNA *sma-miR-10-5p*, from *S. mansoni* is internalized in vertebrate host’s T-cells, where it targets MAP3K7 involved in NF-κB activity regulation, thereby disrupting Th2 lineage and impairing the host’s immune responses (Hamway et al., 2022). Also, the presence of two miRNAs from *S. mansoni*, *sma-miR-bantam* and *sma-miR-10* has been observed *in situ* within the gastrointestinal tract and the mesenteric lymph nodes of its vertebrate murine host during chronic infection (Meningher et al., 2020). Additionally, these miRNAs have also been detected in T lymphocytes (both Th1 and Th2) isolated from exosomes released by adult worms in the serum of infected patients. The success of the parasite or its elimination by the host could therefore also involve parasite miRNAs altering host immune response, or impacting directly the host miRNAs involved in an effective defence against the pathogen (Cai et al., 2015; Zheng et al., 2013; Zhu et al., 2015a; Kifle et al., 2020; Mu et al., 2021). This molecular dialogue between *Biomphalaria* spp. and *Schistosoma* spp. remains to be elucidated even though some indications suggest that following parasite exposure, some parasite miRNAs are present in snails and may interfere with the snail immune response or with snail miRNAs abundance (Phan et al., 2024). For example, 24 h after *S. mansoni* infection in *B. glabrata* snails, 11 mature miRNAs from the parasite were detected, including *sma-miR-2d-3p* and *sma-miR-190-3p* (Portet et al., 2019). Notably, the *sma-miR-190-3p* was found in both sympatric and allopatric conditions and is predicted to target biomphalysin transcript (Portet et al., 2019).

## 4 Conclusions

Herein, we describe the miRNome expressed in the hemolymphatic compartment of the snail *B. glabrata*. This first description should lay the foundation for a deeper understanding of the snail’s immune response to pathogens. With this computational approach, we identified 63 miRNAs of which 25 new miRNAs. Several isomiRs have been founded such as *bgl-miR-92-3p.1.1; bgl-miR-92a-3p.1.2* or *bgl-miR-92a-3p.1.3*. The miR-92 family is highly conserved in animals and known to regulate multiple immune pathways in organisms; it could be a good candidate to investigate the implications in the immunity in *B. glabrata* against its parasite *S. mansoni*. MiRNAs would play a key role in this interaction, drive immune responses and be involved in compatibility polymorphism. The newly identified miRNAs in our study suggest that specific targets related to immune pathways could be regulated by these small RNAs. Indeed, in our study, we predicted *in silico* the potential regulation of immune molecules by miRNAs identified. Humoral factors such as interleukin receptors are predicted to be target by several miRNAs (*bgl-miR-1985-5p; bgl-miR-184-3p* or *bgl-miR-449-5p*) who are highly expressed in hemolymph. Immune molecules such biomphalysins, BgTEP and others have been identified as potential targets for miRNAs with multiples sites of binding. These discoveries could lead to immune regulation mechanisms studies by miRNAs in *B. glabrata*. To further investigate their roles in the snail-parasite interaction, we propose to elucidate their specific functions and effects on snail extreme-selected phenotypes using antagomiR or mimicmiR in snails in response to *S. mansoni*. Knock-down experiments would reveal whether specific miRNAs could be responsible in an efficient immune response or if the parasite’s miRNAs could thwart the snail defence. Taken together, further investigations involving a well-characterized miRNome of the host are essential to determine whether the findings in vertebrate systems also apply to invertebrate systems. Indeed, a key question has been raised as to whether parasite miRNAs might function as xeno-miRNAs to circumvent host immunity. We consider that the *Biomphalaria*-parasite molecular dialogue could play a significant role in their interaction, establishing a specific molecular cross-talk through the modulation of key gene players.

## CRediT authorship contribution statement

**Sarah Dametto:** Conceptualization, Methodology, Investigation, Resources, Data curation, Writing – original draft, Writing – review & editing. **Benjamin Gourbal:** Conceptualization, Writing – original draft, Writing – review & editing. **Cristian Chaparro:** Methodology, Data curation, Writing – review & editing. **Silvain Pinaud:** Conceptualization, Investigation, Writing – review & editing. **David Duval:** Conceptualization, Investigation, Writing – original draft, Writing – review & editing, Supervision, Funding acquisition.

## Ethical approval

Not applicable.

## Funding

This work was funded by BQR (Name: mimicSNAIL) from UPVD. This study is set within the framework of the “Laboratoires d’Excellences (LABEX)” TULIP (ANR-10-LABX-41) and CeMEB (ANR-10-LABX-04-01). The funders had no role in study design, data collection and analysis, decision to publish, or preparation of the manuscript.

## Declaration of competing interests

The authors declare that they have no known competing financial interests or personal relationships that could have appeared to influence the work reported in this paper.

## Data Availability

The data supporting the conclusions of this article are included within the article and its supplementary files.
