# Supplementary material for: Unveiling the hemolymphatic miRNome composition of the schistosomiasis vector snail Biomphalaria glabrata
Source: Curr Res Parasitol Vector Borne Dis. 2025 Apr 22;7:100262. doi: 10.1016/j.crpvbd.2025.100262 (PMC12261956; doi:10.1016/j.crpvbd.2025.100262)
Supplement: Multimedia component 1 [file mmc1.pdf]

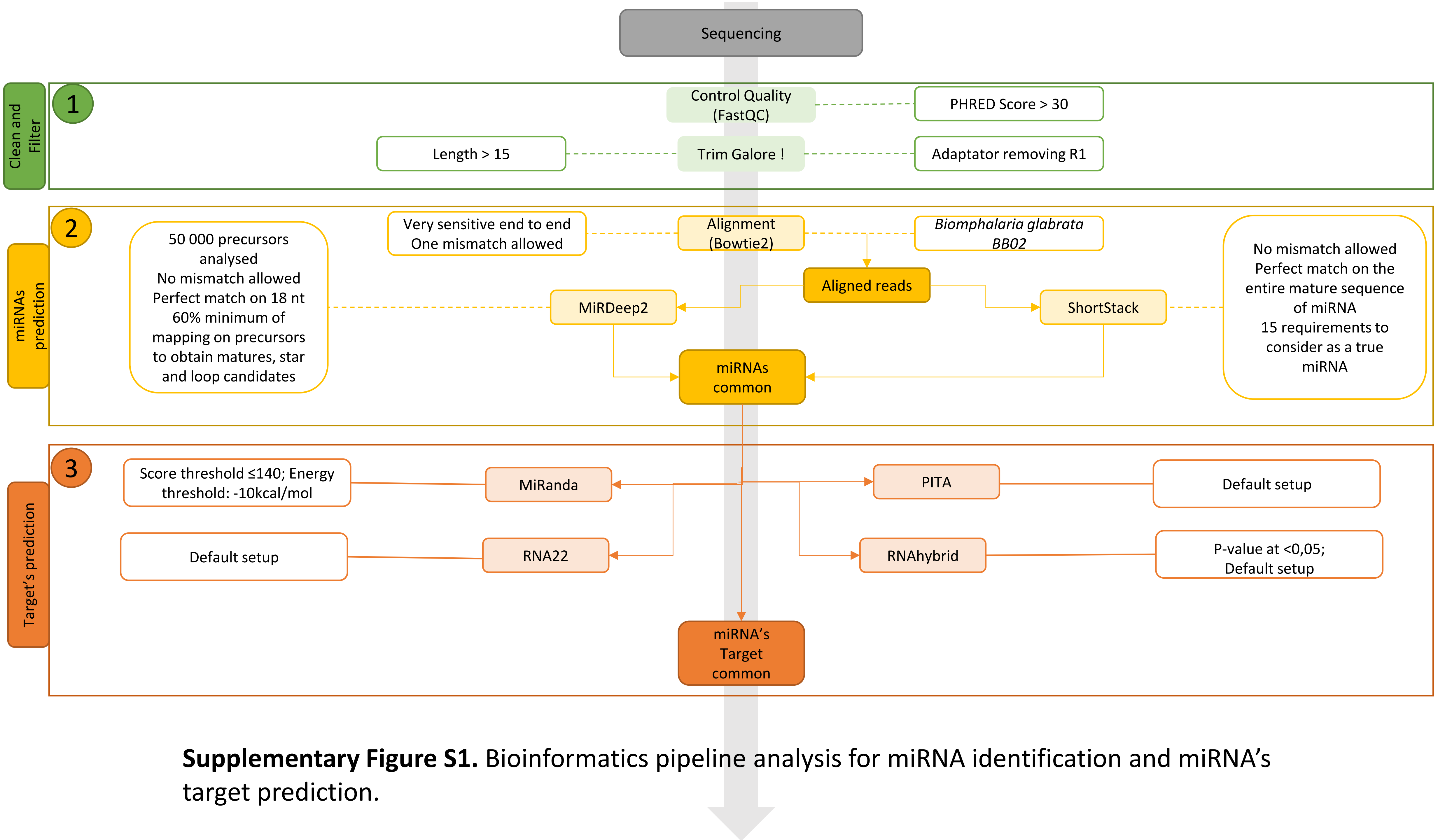

Supplementary Figure S1. Bioinformatics pipeline analysis for miRNA identification and miRNA's target prediction.

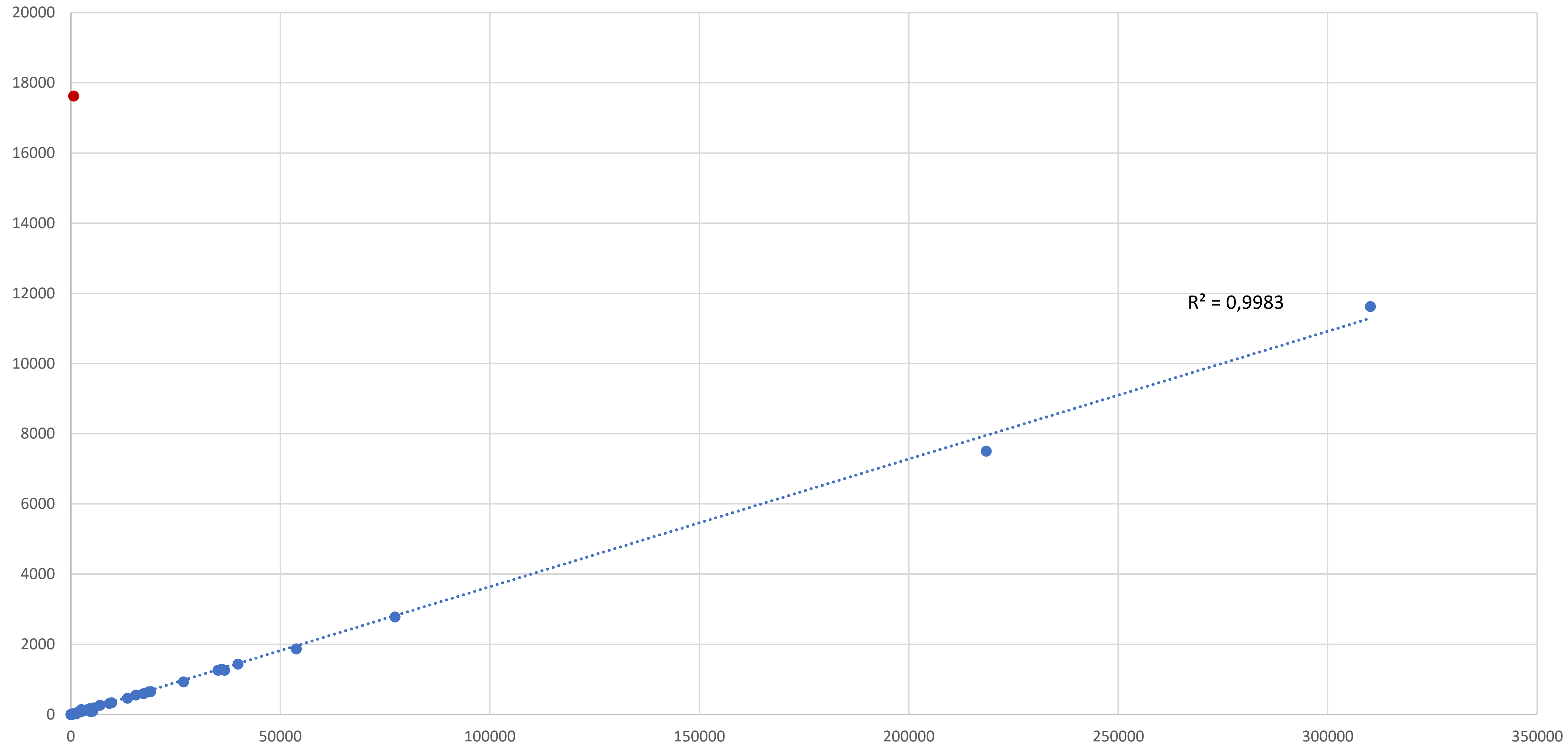

Supplementary Figure S2. Linear regression of normalized RPM of MiRDeep2 and ShortStack for all miRNAs commonly predicted.

The necessity of the calculated RPM for the MiRDeep2 miRNA prediction allowed to normalise the abundance between the two predictions and a regression linear have been done with R^2 at 0.9983

Supplementary Table S1. Distribution of RNA non-coding classes of total reads and unique reads, aligned on genome BB02 of *Biomphalaria glabrata*.

	Unique	Count
Reads (adapter trimmed)	7 536 417	68 000 347
Reads (15-50 nt)	6 504 365	67 431 390
Not matching genome	1 887 218	41 255 826
Kraken bacterial/viruses mapping	816 901	9 450 645
Matching genome	769 104	26 175 564
Matching mature miRNAs	2 658	3 571 069
tRNA	16 949	652 939
rRNA	22 625	758 519
snoRNA	2 822	33 710
mRNA	378 280	2 568 202
Others RNAs	68 590	799 500
Unknown Reads ncRNA of *B.glabrata*	277 180	17 792 171

Note: Others RNAs (RNase MRP RNA; snRNA; scRNA; lncRNA; mRNA; RNase P RNA; SRP RNA; ncRNA, pre-miRNA)

Supplementary Table S2. NCBI number accession and Gene corresponding to TEPs used for analysis

TEPs Name	NCBI reference	BB02 Reference
BgC3-1	MW759477.1	BGLB025256/BGLB021062/BGLB035879/BGLB000085
BgC3-2	MW759478.1	BGLB018444
BgC3-3	MW759479.1	BGLB030610/BGLB020436
BgA2M	MW768931.1	BGLB016521
BgMCR1	MW759480.1	BGLB036531/BGLB035268
BgMCR2	MW759481.1	BGLB022655/BGLB025060/BGLB032488/BGLB026925
BgTEP1	MW759482.1	BGLB028355/BGLB031097/BGLB035738/BGLB021162/BGLB039358
BgTEP2	MW768915.1	BGLB021854
BgTEP3	MW759483.1	BGLB000023/BGLB028857
BgTEP4	MW759484.1	BGLB001162/BGLB036709/BGLB000155/BGLB032760
BgCD109	MW768927.1	BGLB022293/BGLB031746/BGLB021085

Supplementary Table S3. NCBI number accession and Gene corresponding to FREPs used for analysis

FREPs Name	NCBI reference	BB02 Reference
FREPH	KY024265.1	BGLB000019
FREPL	KY024266.1	BGLB000140
FREPI2	KY024263.1	BGLB000096
FREPC1	KY024246.1	BGLB028617
FREPB	KY024245.1	BGLB000179
FREPJ5	KY024264.1	BGLB005224
FREPJ3	KY024257.1	BGLB040226
FREPJ1	KY024254.1	BGLB032905
FREPJ2	KY024252.1	BGLB000019
FREPJ4	KY024251.1	BGLB000074
FREI3	KY024248.1	BGLB029167
FREPJ1 Precursor	KY024242.1	BGLB000021
FREPM	KY024241.1	BGLB007576
FREPA	KY024240.1	BGLB000141

Supplementary Table S4. Fifteen genes (BB02 and Lab' annotation) predicted and targeted on their UTR and Protein Coding Gene by miRNAs

Gene ID	Gene annotation	Hybridation localization	miRNAs
**BGLB000051**	baculoviral IAP repeat-containing protein 2-like isoform	**3UTR**	bgl-miR-33-3p; bgl-miR-71-5p
		**5UTR**	bgl-miR-bantam-1-3p; bgl-miR-bantam-2-3p
		**PCG**	bgl-miR-981-3p; bgl-miR-bantam-1-3p; bgl-miR-bantam-2-3p; bgl-miR-let-7-5p; bgl-miR-449-5p
**BGLB000786**	ATP-binding cassette sub-family D member 2-like	**3UTR**	bgl-miR-449-5p
		**5UTR**	bgl-miR-71-5p
		**PCG**	bgl-miR-1994a-3p; bgl-miR-1994b-3p; bgl-miR-2001-5p; bgl-miR-2c-3p; bgl-miR-71-5p; bgl-miR-22707-5p; bgl-miR-87b-1-3p; bgl-miR-87b-2-3p; bgl-miR-bantam-1-3p; bgl-miR-bantam-2-3p; bgl-miR-9-5p
**BGLB000846**	Not Annotated	**3UTR**	bgl-miR-33-3p; bgl-miR-87b-1-3p; bgl-miR-87b-2-3p
		**5UTR**	bgl-miR-22707-5p
		**PCG**	bgl-miR-10a-5p; bgl-miR-277a-3p; bgl-miR-29-3p; bgl-miR-449-5p
**BGLB000876**	ras guanine nucleotide exchange factor L-like	**3UTR**	bgl-miR-11705-5p; bgl-miR-12604-5p
		**5UTR**	bgl-miR-279-3p
		**PCG**	bgl-miR-2f-3p; bgl-miR-449-5p; bgl-miR-71-5p; bgl-miR-12604-5p; bgl-miR-let-7-5p
**BGLB001150**	receptor-type tyrosine-protein phosphatase kappa-like isoform	**3UTR**	bgl-miR-263b-5p
		**5UTR**	bgl-isomiR-92-3p.1.1
		**PCG**	bgl-miR-133-3p; bgl-isomiR-2c-3p.1; bgl-miR-2001-5p; bgl-miR-216b-5p; bgl-miR-29-3p; bgl-miR-2c-3p; bgl-miR-31-5p; bgl-miR-449-5p; bgl-miR-750-3p; bgl-miR-12604-5p; bgl-miR-9-5p
**BGLB001225**	transcriptional activator Myb-like isoform	**3UTR**	bgl-miR-9-5p
		**5UTR**	bgl-miR-bantam-1-3p; bgl-miR-bantam-2-3p
		**PCG**	bgl-miR-190-5p; bgl-miR-12604-5p; bgl-miR-9-5p
**BGLB016762**	Synaptotagmin 7	**3UTR**	bgl-miR-9-5p
		**5UTR**	bgl-miR-2c-3p
		**PCG**	bgl-miR-981-3p; bgl-miR-9-5p
**BGLB017474**	bridge-like lipid transfer protein family member 1 isoform	**3UTR**	bgl-miR-33-3p
		**5UTR**	bgl-miR-745b-3p
		**PCG**	bgl-miR-10a-5p; bgl-isomiR-1175-3p.1.1; bgl-miR-125-5p; bgl-miR-133-3p; bgl-miR-184-3p; bgl-miR-190-5p; bgl-miR-1985-5p; bgl-miR-1994a-3p; bgl-miR-1994b-3p; bgl-miR-2001-5p; bgl-miR-216b-5p; bgl-mir-252a-5p; bgl-isomiR-92a-3p.1.3; bgl-miR-279-3p; bgl-miR-281-5p; bgl-miR-2c-3p; bgl-miR-31-5p; bgl-miR-375-3p; bgl-miR-449-5p; bgl-miR-71-5p; bgl-miR-12097-1-3p; bgl-miR-12097-2-3p; bgl-miR-745b-3p; bgl-miR-750-3p; bgl-miR-12604-5p; bgl-miR-22707-5p; bgl-miR-87b-1-3p; bgl-miR-87b-2-3p; bgl-miR-92a-3p; bgl-miR-96a-5p; bgl-miR-981-3p; bgl-miR-bantam-1-3p; bgl-miR-bantam-2-3p; bgl-miR-let-7-5p; bgl-miR-9-5p
**BGLB020718**	integrator complex subunit 13-like	**3UTR**	bgl-miR-71-5p; bgl-miR-9-5p
		**5UTR**	bgl-miR-29-3p; bgl-miR-31-5p; bgl-miR-22707-5p
		**PCG**	bgl-miR-1985-5p; bgl-miR-1994a-3p; bgl-miR-1994b-3p; bgl-miR-31-5p; bgl-miR-375-3p; bgl-miR-449-5p; bgl-miR-71-5p; bgl-miR-745b-3p; bgl-miR-8-3p; bgl-miR-let-7-5p; bgl-miR-9-5p
**BGLB022562**	verprolin-like	**3UTR**	bgl-miR-1985-5p; bgl-miR-9-5p
		**5UTR**	bgl-miR-12097-1-3p; bgl-miR-12097-2-3p; bgl-miR-9-5p
		**PCG**	bgl-miR-184-3p; bgl-miR-281-5p; bgl-miR-449-5p; bgl-miR-let-7-5p; bgl-miR-9-5p
**BGLB027526**	adhesion G-protein coupled receptor D1-like	**3UTR**	bgl-miR-2001-5p
		**5UTR**	bgl-miR-let-7-5p
		**PCG**	bgl-miR-71-5p; bgl-miR-9-5p
**BGLB028227**	D(2) dopamine receptor A-like isoform	**3UTR**	bgl-miR-279-3p
		**5UTR**	bgl-miR-184-3p; bgl-miR-190-5p; bgl-miR-33-3p
		**PCG**	bgl-miR-449-5p
**BGLB030287**	ankyrin repeat and KH domain-containing protein 1-like	**3UTR**	bgl-miR-11705-5p
		**5UTR**	bgl-miR-2c-3p
		**PCG**	bgl-miR-449-5p; bgl-miR-745b-3p; bgl-miR-12604-5p
**BGLB036645**	RWD domain-containing protein 2A	**3UTR**	bgl-miR-96a-5p
		**5UTR**	bgl-miR-2f-3p
		**PCG**	bgl-miR-10a-5p; bgl-miR-745b-3p; bgl-miR-12604-5p
**BGLB038139**	gem-associated protein 5-like	**3UTR**	bgl-miR-22707-5p
		**5UTR**	bgl-isomiR-92a-3p.1.3; bgl-miR-92a-3p; bgl-isomiR-92a-3p.1.2
		**PCG**	bgl-miR-1985-5p; bgl-miR-12604-5p; bgl-miR-22707-5p; bgl-miR-9-5p
